# Association analysis of germline variants in GEN1 with a susceptibility to prostate cancer in Polish men

**DOI:** 10.1186/s13053-026-00343-0

**Published:** 2026-06-10

**Authors:** Katarzyna Gliniewicz, Klaudia Stempa, Dominika Wokołorczyk, Wojciech Kluźniak, Milena Kiljańczyk, Helena Rudnicka, Tomasz Huzarski, Jacek Gronwald, Jan Uciński, Tadeusz Dębniak, Marta Grabarczyk, Anna Jakubowska, Marek Szwiec, Marcin Lener, Jan Lubiński, Steven A. Narod, Mohammad R. Akbari, Cezary Cybulski

**Affiliations:** 1 International Hereditary Cancer Center, Department of Genetics and Pathology, Pomeranian Medical University in Szczecin, Unii Lubelskiej 1, Szczecin, 71-252 Poland; 2 Department of Clinical Genetics and Pathology, University of Zielona Góra, Zielona Góra, Poland; 3 Independent Laboratory of Molecular Biology and Genetic Diagnostics, Pomeranian Medical University, Szczecin, Poland; 4 Clinics of Oncology, University Hospital in Zielona Góra, Zielona Góra, Poland; 5 Women’s College Hospital, Women’s College Research Institute, University of Toronto, Toronto, Canada; 6 Dalla Lana School of Public Health, University of Toronto, Toronto, Canada

**Keywords:** *GEN1*, Mutation, Variant, Sequencing, Hereditary, Prostate cancer

## Abstract

**Background:**

The *GEN1* gene is involved in DNA damage repair, as are several prostate cancer susceptibility genes, and is now included in several NGS clinical testing panels. The aim of our study was to investigate the role of *GEN1 *mutations in the etiology of prostate cancer.

**Methods:**

First, we sequenced *GEN1* in 390 Polish men with familial prostate cancer and 300 population controls using exome sequencing to identify protein-truncating variants and to analyze their possible association with prostate cancer risk. In addition, we performed a large association study of a recurrent frameshift variant of *GEN1* (c.1929_1932delAAAG) among 5,857 men with unselected prostate cancer and 3,956 controls. We compared the clinical characteristics of prostate tumors in carriers of c.1929_1932delAAAG and in non-carriers. We conducted loss of heterozygosity (LOH) analysis at the *GEN1* locus in prostate cancers from two men with the c.1929_1932delAAAG variant. To analyze survival, patients were followed from diagnosis to death for an average of 101 months.

**Results:**

We detected two frameshift variants (c.2515_2519delAAGTT (p. Lys839Glufs*2), c.1539delT (p. Asp514Ilefs*13)) by sequencing the *GEN1* gene of 390 Polish patients with familial prostate cancer and 300 cancer-free controls. Neither variant was associated with increased prostate cancer risk: c.2515_2519delAAGTT was detected in 20.5% of 390 cases and in 17.1% of 300 controls (p = 0.28), c.1539delT was detected in one case (0.3%) and none of the controls (p = 1.00). The *GEN1* variant, c.1929_1932delAAAG (p. Lys645Cysfs*29), was observed in 13 of 5,857 (0.22%) unselected cases and 8 of 3,956 (0.20%) controls (OR = 1.10, p = 0.84). Clinical characteristics of prostate tumors in 13 carriers of c.1929_1932delAAAG and 5,844 non-carriers were similar. All-cause survival was similar for variant carriers and non-carriers (age-adjusted HR = 0.68, p = 0.76). The wild-type *GEN1* allele was not lost in two prostate tumors in men with the c.1929_1932delAAAG variant.

**Conclusions:**

Our analysis suggests that *GEN1* is not a prostate cancer susceptibility gene. The study has clinical implications for genetic counseling of men who tested positive for germline variants of *GEN1*.

## Introduction

Prostate cancer (PC) is the most prevalent cancer among men worldwide [1]. The heritability of prostate cancer, based on twin studies, is about 50% [2]. 10% of all prostate cancer cases are familial [3]. Highly penetrant genes are responsible for approximately 5–10% of all prostate cancers [4]; these include *BRCA2* and *HOXB13* [5, 6]. Mutations in *BRCA1*, *CHEK2*, *NBN*, *BRIP1*, *ATM*, and *SPOP* also predispose men to PC, but the associated risks are moderate and less well established [7–10]. Mutations in genes for Lynch and Li–Fraumeni syndromes may confer increased prostate cancer risk [11, 12]. There are also genes associated only with aggressive disease (e.g., *PALB2*) and genes for which mutations were identified in metastatic prostate cancer (e.g., *RAD51C-D* and Fanconi anemia genes).

Genomic instability is a common feature of human cancers. The DNA damage signaling pathway plays a crucial role in maintaining genome integrity and in the pathogenesis of prostate cancer [13]. Many prostate cancer susceptibility genes are involved in the damage repair pathway, including *BRCA2*, *BRCA1*, *CHEK2*, *NBN*, *ATM*, *BRIP1* and *PALB2* [7–10, 14]. The major function of this pathway is the repair of double-strand DNA breaks by homologous recombination using the normal homologous chromosome as a template [15–16]. DNA intermediates, known as Holliday junctions (HJs), are formed during this process. Resolution of HJs is necessary for proper chromosome segregation and DNA repair. When repair is completed, HJs are broken, and two double-stranded DNA particles are created [17–18].

The *GEN1* (OMIM Number: 612449) is implicated in DNA damage repair. It encodes a protein that is a member of the Rad2/XPG family of endonucleases and plays a key role in resolving Holliday junctions (HJs) during DNA repair by homologous recombination [19]. GEN1 also shows activity against structures such as 5′-flaps and replication forks, and it has been suggested to maintain centrosome integrity [20–21]. Given its critical role in DNA repair, it is a candidate prostate cancer susceptibility gene.

It has not been established if mutations in *GEN1* confer an increased risk of prostate cancer. To analyze the contribution of *GEN1* to prostate cancer, we sequenced *GEN1* in 390 Polish men with familial prostate cancer and 300 population controls (by exome sequencing) to identify protein-truncating variants of *GEN1* and to analyze if they are associated with prostate cancer risk (by comparing their frequency between cases and controls). In addition, we performed a large association study of a rare recurrent frameshift variant of *GEN1*, c.1929_1932delAAAG (which was previously identified in the Polish population [22]) among 5,857 men with unselected prostate cancer and 3,956 cancer-free males. We compared the clinical characteristics of prostate tumors in carriers of c.1929_1932delAAAG versus those in non-carriers. We also conducted loss of heterozygosity (LOH) analysis at the *GEN1* locus in PC from two men with the c.1929_1932delAAAG variant.

## Materials and methods

### Patients

We studied two non-overlapping series of men with PC: 390 men with familial prostate cancer (first series) and 5,857 men with unselected prostate cancer (second series).

The first series of cases was derived from a registry of familial cases housed at the Hereditary Cancer Center in Szczecin, Poland. We selected families with strong aggregations of prostate cancer and those with the youngest age of onset in probands and their relatives. The mean number of prostate cancers per family was 2.8. The mean age at diagnosis among the 390 men was 61.6 years (range 38–82). The series has been described previously [23].

The second series of cases included 5,857 men with PC who were diagnosed between 1999 and 2015 in 14 centers situated throughout Poland. This study was initiated in Szczecin in 1999 and was extended to include Białystok and Olsztyn in 2002 and Opole in 2003. Other centers began recruiting between 2005 and 2008 (Koszalin, Gdańsk, Lublin, Łodź, Warszawa, Wrocław, Poznań, Rzeszów, Bydgoszcz, Zabrze). Men with newly diagnosed prostate cancer were invited to participate. Prostate cancer cases enrolled in the study are unselected for age, clinical characteristics (stage, grade, PSA level at diagnosis), family history, or treatment. Study subjects were asked to participate at the time of diagnosis. The patient participation rate was 85.4%. Patients who provided a blood sample within six months of diagnosis were enrolled. Men who provided a blood sample more than 6 months after diagnosis were excluded. The mean age of diagnosis was 67.3 years (range 35–96 years). A family history was obtained by constructing a family tree or completing a standardized questionnaire. All first- and second-degree relatives with prostate cancer, and their ages of diagnosis, were recorded. 711 men reported at least one first- or second-degree relative with prostate cancer (familial cases from unselected cases). In addition, information was obtained on PSA level at the time of diagnosis, grade (Gleason score), and stage, where possible. The vital status (dead or alive) and the date of death of the cases were requested from the Polish Ministry of the Interior and Administration in June 2016 and were obtained in July 2016. Survival data were obtained for all men with prostate cancer.

### Controls

We included two non-overlapping control groups. The first series consisted of 300 Polish cancer-free adults, 155 women (age range 40–84; mean 56.9) and 145 men (age range 45–89; mean 62.1), who underwent exome sequencing in a previous study, and were used as a reference group. It is described in detail previously [23]. This series was used to estimate the frequencies of *GEN1* variants in the underlying Polish population and was used as a reference to assess the association of *GEN1* variants detected by sequencing men with familial prostate cancer.

The second control group consisted of 3,956 cancer-free men aged 23 to 90 years (mean age, 61.2 years) from the International Hereditary Cancer Center (IHCC) cohort (Szczecin, Poland) [24]. This large control group was used as a reference in the association study of the c.1929_1932delAAAG variant with prostate cancer risk in the Polish population (which included 5,857 unselected prostate cancer cases and 3,956 controls).

All cases and controls were ethnic Poles. All individuals signed informed consent for genetic testing. The study was approved by the Ethics Committee of the Pomeranian Medical University in Szczecin (IRB No. KB-0012/97/17).

## Methods

### Sequencing of GEN1

We analyzed the entire coding sequence of *GEN1* from the whole exome sequencing (WES) data of 390 men with hereditary prostate cancer and 300 controls. Germline DNA was isolated from peripheral blood leukocytes using standard methods. The Agilent SureSelect Human Exome Kit (V6) was used to capture target regions. The kit captures 64 Mbp (2.2%) of the human genome, covers coding exons in CCDS and RefSeq databases, as well as exons annotated by the GENCODE project. This includes ~ 205,000 exons in ~ 35,000 genes, including protein-coding and non-coding RNA genes.

The captured regions for each sample were barcoded, and every 16 samples were pooled and used for paired-end sequencing for 150 cycles (generating 150 bp reads) on a high-throughput sequencing cartridge of the Illumina NextSeq 500.

The sequence reads for each exome sequence were aligned to the reference sequence of the human genome using the Burrows-Wheeler Aligner. The mean depth of coverage was approximately 100x (range 52x to 154x). On average, 97.4% (range 91.2% to 99.1%) of the CCDS exons were covered at 20x or higher depth of coverage and used for variant calling. For *GEN1*, on average, 99.5% of its coding exons (exons 2–14, NM_182625.4) were covered at 20x or more.

The Picard package (http://picard.sourceforge.net) was used to convert the SAM files to BAM format and sort and index the BAM files. In the next step, all the unmapped reads aligned to more than one human genome region and all duplicate reads were filtered out from the BAM file using the GATK package. Exact duplicate reads were collapsed to avoid inflated coverage. The HaplotypeCaller module of the GATK package was used for calling both SNPs and indels. Regions with at least 20-fold depth of coverage were used for calling variants, and a different nucleotide from the reference sequence seen in at least 25% of the reads aligned to a given position was called as a variant. The SNP & Variation Suite (GoldenHelix Inc., Bozeman, MT, USA) was used for annotating called variants and to determine the effect of *GEN1* variants on the coding protein. We searched for loss-of-function variants, including frameshift indels, nonsense mutations, splicing-site variants, and start-codon-loss mutations [23].

### Genotyping

Genomic DNA was isolated from 5 to 10 mL of peripheral blood by the non-enzymatic method [25]. The c.1929_1932delAAAG (p.Lys645Cysfs*29) variant was genotyped using a TaqMan assay (Thermo Fisher Scientific, Waltham, MA, USA) on a LightCycler Real-Time PCR 480 System (Roche Life Science, Mannheim, Germany) using probes: [HEX]ACACTGCAAACATAAAGAAAGT[BHQ1] and [6FAM]TACACTGCAAACATAAAGTGTT[BHQ1] and primers: forward- AAAATATCCCAGAACAACTGTCCTG and reverse- CTTCAGGACTAATCCCATCAGAATC. Real-time PCR conditions are described previously [26]. All variants were confirmed by Sanger sequencing. Sequencing reactions were performed using a BigDye Terminator v3.1 Cycle Sequencing Kit (Thermo Fisher Scientific) according to the manufacturer’s protocol using forward primer CCAGAACAACTGTCCTGTGA and reverse primer TGCTCTTCAGGACTAATCCCA. Sequencing products were analyzed on an ABI Prism 3100 Genetic Analyzer (Thermo Fisher Scientific).

### Loss of heterozygosity analysis

Formalin-fixed paraffin-embedded (FFPE) prostate cancer tissue samples from two *GEN1* mutation carriers were available in the Department of Pathology of Pomeranian Medical University in Szczecin. A pathological review of these samples was conducted by a pathologist. Loss of heterozygosity (LOH) analysis at the *GEN1* locus was performed in DNA from micro-dissected tumors from two *GEN1* variant-positive men (p.Lys645Cysfs*29) as described previously [27] with some modification: (1) DNA was isolated with QIAamp DNA FFPE Tissue Kit (from QIAGEN); (2) LOH was analyzed by direct Sanger sequencing of a 92 bp DNA fragment containing the p.Lys645Cysfs*29 variants (forward primer CCAGAACAACTGTCCTGTGA and reverse primer TGCTCTTCAGGACTAATCCCA).

In brief, the pathologist (J.L.) selected fields of tissue containing > 80% prostate cancer cells on H&E (Hematoxylin and Eosin)-stained slides using a light microscope. Then, he marked these areas on FFPE blocks, took tissue cores from the marked fields, and provided the cores for DNA isolation. DNA samples were amplified by Sanger sequencing. As controls for the LOH study, we used five germline DNA samples clearly heterozygous for the founder *GEN1* variant, which were isolated from blood samples of the variant carriers.

**Fig. 1:**
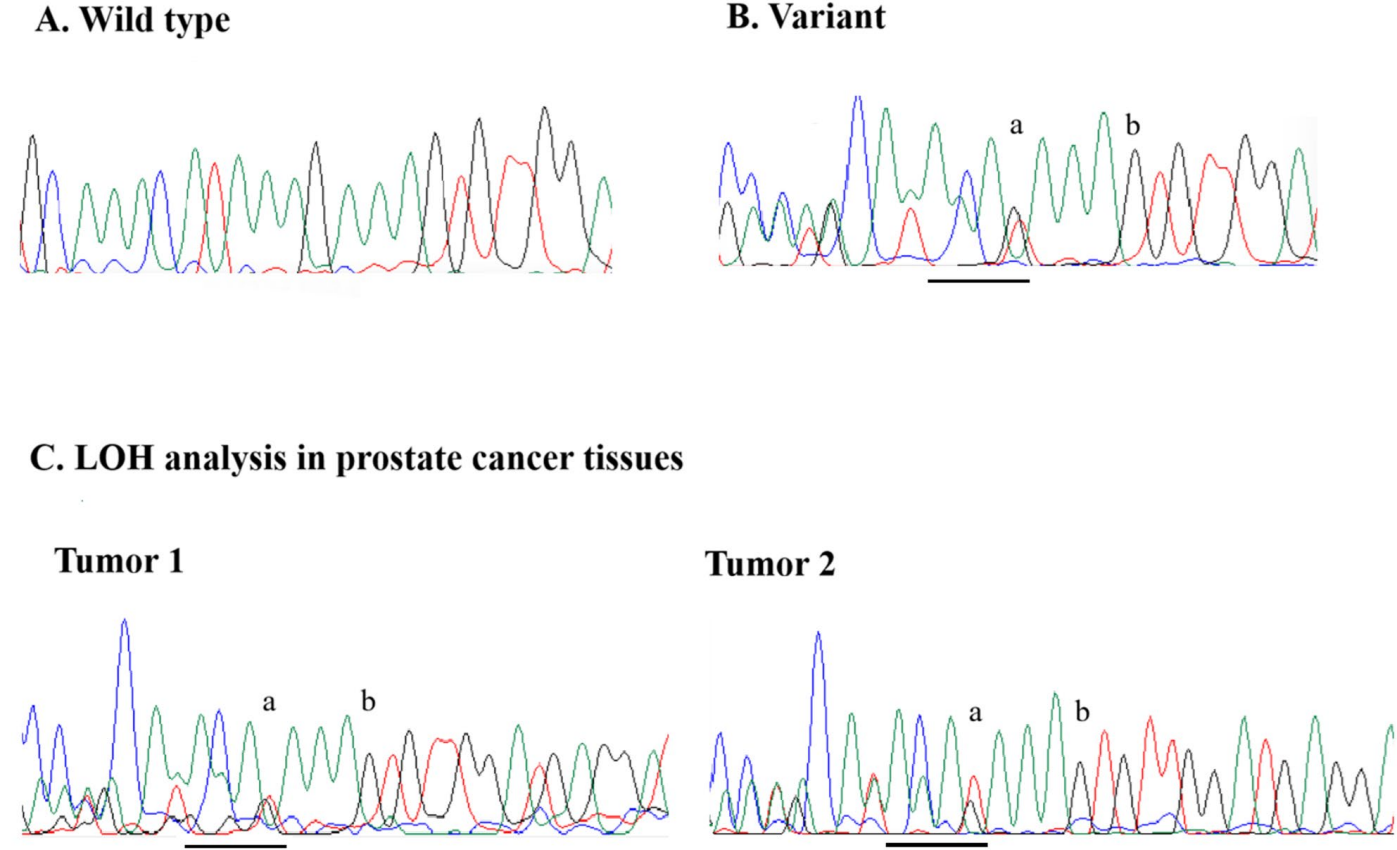
Sequencing chromatograms (with the reverse primer): (**A**) Wild type sequence (sequencing of germline DNA from individual without p.Lys645Cysfs*29) (**B**) *GEN1* p.Lys645Cysfs*29 variant (example of sequencing of germline DNA from deletion carrier); (**C**) Loss of heterozygosity (LOH) analysis in prostate cancers from two carriers of p.Lys645Cysfs*29; retention of the *GEN1* wild type allele is observed in all cancers (peaks corresponding to nucleotides G, used in loss of the wild type analysis (to produce a/b ratio) are presented by letters “a” and “b”). A 4-bp deletion of *GEN1* is underlined. Green peaks correspond to nucleotide A, red to nucleotide T, black to nucleotide G, and blue to nucleotide C

Given that a series of tissues including > 80% cancer cells were chosen, we expect that the complete loss of the wild-type allele would lead to about 80% reduction of the signal from the normal allele on sequencing chromatograms at the site of the small deletion. To evaluate whether the wild type allele is lost in cancer tissues, we calculated the ratio between the peak height of the first deleted nucleotide - nucleotide G (investigated peak) corresponding to the wild type allele (marked by “a” on Fig. 1), to the height of the control peak corresponding to the first nucleotide G located 3 bp 5’ to the deletion (marked by “b” on Fig. 1) for two prostate cancers and five controls (as mentioned above).

### Statistical analysis

The prevalence of two *GEN1* variants (c.1539delT and c.2515_2519delAAGTT) was estimated in 390 familial prostate cancer cases and 300 controls who were fully sequenced. The prevalence of the c.1929_1932delAAAG allele (third investigated variant) was estimated in 5,857 unselected prostate cancer cases and 3,956 cancer-free men. Odds ratios were generated from two-by-two tables. Statistical significance was assessed using the Fisher exact test or the Chi-squared test, where appropriate.

Men with prostate cancer, with and without the c.1929_1932delAAAG variant, were compared for age at diagnosis, family history, and clinical features of the prostate cancers. Statistical significance was assessed using the Fisher exact test or the Chi-squared test, where appropriate. Means were compared using a t-test. Medians were compared using the Mann-Whitney test.

The survival of men with and without the c.1929_1932delAAAG variant was followed from the date of diagnosis until death or until July 2016. To estimate 5- and 10-year survival, Kaplan-Meier curves were constructed for variant carriers and non-carriers. HR for mortality and significance for the c.1929_1932delAAAG variant were calculated using Cox regression. All analyses were performed using MedCalc Version 23.2.1.

## Results

We identified two frameshift *GEN1* variants in 390 Polish men familial prostate cancer and 300 controls who were sequenced. Both were small deletions located in the last *GEN1* exon (Table 1).

**Table 1:** *GEN1* truncating variants detected by sequencing among Polish patients with familial prostate cancer from families with strong aggregation of this cancer and in controls

Variant	Exon	Genotype	Frequency among cases	Frequency among controls	*P* value
c.1539delT (p.Asp514Ilefs*13)	13	del/wt	1/380 (0.3%)	0/299 (0.0%)	1.00
c.2515_2519delAAGTT	13	del/del	4/380 (1.1%)	4/299 (1.3%)	0.74
(p.Lys839Glufs*2)		del/wt	78/380 (20.5%)	51/299 (17.1%)	0.28
		del/del or del/wt	82/380 (21.6%)	55/299 (18.4%)	0.34

Nucleotide positions based on the NM_182625.4 transcript of *GEN1*; del-deletion, wt -wild type.

The first was a common variant (c.2515_2519delAAGTT, p. Lys839Glufs*2). In homozygous or heterozygous state, it was seen in 21.6% of 380 patients and in 18.4% of 299 controls (*p* = 0.74). In the homozygous state, it was present in 1.1% of cases and 1.3% of controls (*p* = 0.74); in the heterozygous state, it was detected in 20.5% of cases and 17.1% of controls (*p* = 0.28). The c.2515_2519delAAGTT deletion was common and detected with similar frequency in cases and controls, so it was not further studied. The second variant was rare (c.1539delT, p. Asp514Ilefs*13). It was detected in one case (0.3%) and 0% of controls who were sequenced (*p* = 1.00). The c.1539delT deletion was non-recurrent; it was not associated with prostate cancer in our sequencing series (*p* = 1.00), so it was not further analyzed.

Previously, we identified another (third) *GEN1* recurrent frameshift variant c.1929_1932delAAAG (p. Lys645Cysfs*29) in the Polish population [22], and now we wished to establish whether it predisposes to prostate cancer. The variant was now genotyped in 5,857 men with unselected prostate cancer and 3,956 cancer-free male controls. It was observed in 13 of 5,857 unselected cases (0.22%) and 8 of 3,956 (0.20%) controls (OR = 1.10, *p* = 0.84). The variant was found in two of 717 (0.28%) familial prostate cancer cases among the unselected cases (OR = 1.38, *p* = 0.68).

We compared the clinical characteristics of cases with prostate cancer with and without the *GEN1* c.1929_1932delAAAG deletion. We did not observe a significant difference in age at diagnosis between variant carriers and non-carriers (mean age of onset was 70.3 versus 67.3, respectively; *p* = 0.19). Also, PSA at diagnosis, Gleason score, and tumor stage were similar in men with the variant compared with those without the deletion (Table 2). However, the small number of variant carriers limits the power of this comparison.

**Table 2:** Clinical characteristics of prostate cancers in men with the GEN1 c.1929_1932delAAAG (p. Lys645Cysfs*29) variant and non-carriers

Group		c.1929_1932del positive cases (*n* = 13)	c.1929_1932del negative cases (*n* = 5844) *	*p*-value
Age of diagnosis	mean (range)	70.3 (56–81)	67.3 (35–93)	0.19
	< 60	23.1% (3/13)	18.5% (1080/5844)	0.95
	61–70	23.1% (3/13)	44.4% (2595/5844)	0.21
	> 70	53.8% (7/13)	37.1% (2093/5844)	0.29
PSA at diagnosis	median (range)	10.5 (2.9–35.4)	10.7 (0.1–5000)	0.86
	≤ 4.0	10.0% (1/10)	5.9% (155/2634)	0.58
	4.1–10	40.0% (4/10)	41.6% (1097/2634)	0.92
	10.1–20.0	40.0% (4/10)	24.6% (644/2634)	0.48
	> 20.0	10.0% (1/10)	27.9% (738/2634)	0.36
Gleason score	< 7	45.4% (5/11)	54.0% (1649/3053)	0.79
	7	36.4% (4/11)	27.9% (851/3053)	0.77
	> 7	18.2% (2/11)	18.1% (553/3053)	1.00
Stage	T1/2	77.8% (7/9)	75.2% (1722/2289)	0.86
	T3/4	22.2% (2/9)	24.8% (567/2289)	0.86
Positive family history	positive	15.4% (2/13)	15.3% (715/4661)	0.99
Deceased men	proportion	30.8% (4/13)	41.8% (2441/5844)	0.60

Data represent mean (range) or number/total (%), p-values compare variant-positive to variant-negative patients, calculated by Fisher’s exact test.

All-cause survival was available for 5,857 prostate cancer patients. During the median follow up of 101 months, there were 4 deaths in 13 (30.8%) carriers of c.1929_1932delAAAG and 2441 deaths among 5,844 non-carriers (41.8%,), but the difference was not significant (*p* = 0.60). The 5-year survival was 76% in carriers and 72% in non-carriers. The 10-year survival was 66% in carriers and 55% in non-carriers. The age-adjusted HR for mortality associated with the c.1929_1932delAAAG variant was 0.68 (95%CI 0.25 to 1.80; *p* = 0.76; Cox regression).

We analyzed tumor DNA samples from prostate cancers of two carriers with the *GEN1* p. Lys645Cysfs*29 variants for loss of heterozygosity at the *GEN1* locus. To investigate if the wild type allele is lost in cancer tissues, we calculated the ratio between the peak height of the first deleted nucleotide - nucleotide G corresponding to the wild type allele (marked by “a” on Fig. 1), to the height of the control peak corresponding to the first nucleotide G located 3 bp 5’ to the deletion (marked by “b” on Fig. 1). For five germline DNA samples of heterozygous *GEN1* variant carriers (controls), the peak ratios a/b were from 0.43 to 0.51. For two prostate cancer tissues from p. Lys645Cysfs*29 carriers, the ratios were 0.46 and 0.49, suggesting that the wild-type allele was not lost (Fig. 1). It is consistent with the observation that the signal of nucleotide T specific for the deletion (red peak marked by “a” on Fig. 1) is strong in both tumors, and it should be lost in the case of LOH.

## Discussion

*GEN1* is a key gene in the homologous recombination repair of DNA breaks. It is located on chromosome 2p24.2. Its coding sequence has 13 exons and encodes a protein of 908 amino acids in length, which includes three functional domains: N-terminal and internal xeroderma pigmentosum group G nuclease domains (the XPG-N and XPG-I), and a helix-hairpin-helix domain [28, 29]. The *GEN1* gene is involved in DNA damage repair, as several prostate cancer susceptibility genes (i.e., *BRCA2*, *BRCA1*, *CHEK2*, *NBN* and *ATM*) [23].

To date, little is known about cancer susceptibility in carriers of *GEN1* mutations. The commonly studied is the association between *GEN1* and breast cancer; current evidence suggests that *GEN1* variants do not confer an increased risk of breast cancer [22, 30, 31]. The association between *GEN1* and prostate cancer is less well studied. In a UK-based cohort of 1281 young-onset prostate cancer cases (diagnosed at ≤ 60 years) and 1160 selected controls, two *GEN1* truncating variants were detected among cases and none in controls [32]. In the most recent and largest study of men of European ancestry with prostate cancer, 5 *GEN1* deleterious variants were detected in 2397 men with metastatic disease (OR = 2.1, *p* = 0.26), 14 in 9185 men with aggressive prostate cancer (OR = 0.7, *p* = 0.45), compared to 17 in 8361 patients with non-aggressive prostate cancer [33]. No other studies to date have analyzed the influence of *GEN1* variants on prostate cancer risk.

In this study, we detected two frameshift variants (p. Lys645Cysfs*29 and p.Asp514Ilefs*13) by sequencing the *GEN1* gene of 390 Polish patients with familial prostate cancer and 300 cancer-free controls. One was a common small deletion, c.2515_2519delAAGTT (p. Lys839Glufs*2), that causes protein truncation, removing 9% of GEN1 3′-end sequence. The variant was present with a similar frequency in 390 Polish familial prostate cancer cases and 300 controls, suggesting that it is not associated with an increased risk of prostate cancer. Previously, it was found not to be associated with breast cancer [22, 30]. Another variant was a non-recurrent *GEN1* small deletion c.1539delT (p. Asp514Ilefs*13) that removes 42% of its coding sequence. It was detected only in one of 390 Polish familial prostate cancer cases and in none of 300 controls (*p* = 1.00). Although our study suggests that it is not associated with prostate cancer risk, the variant was rare, and further studies are needed.

The last variant we investigated is a rare recurrent deletion, c.1929_1932delAAAG, previously detected in our population [22]. The variant creates a premature stop codon (p. Lys645Cysfs*29) in *GEN1*. It is expected to disrupt the last 29% of the amino acids of GEN1. Prediction algorithms are not available, and its functional significance is unknown. Because the variant was rare and recurrent, we decided to analyze whether it is implicated in the etiology of prostate cancer in a large association study of 5,857 unselected cases and 3,956 controls. We analyzed whether prostate cancers with and without the variant were different in their clinical characteristics. We evaluated the loss of heterozygosity at the *GEN1* locus in prostate cancers from two carriers of the c.1929_1932delAAAG deletion. In these analyses, we did not see evidence of susceptibility to prostate cancer among carriers of the c.1929_1932delAAAG variant. However, this variant was rare, and a modest increase in prostate cancer risk given the variant cannot be excluded.

GEN1, a member of the Rad2/XPG nuclease family, was isolated recently from human cells and shown to promote HJ resolution in vitro and in vivo. GEN1 protein contains the XPG-N, XPG-I, and helix–hairpin–helix domains essential for its function and C-terminal tail [34]. The XPG and helix–hairpin–helix domains are localized in the highly conserved region of GEN1 over the first 480 amino acids [35]. We did not detect any truncating mutation in the conserved region of the first 480 codons of *GEN1*. Protein-truncating mutations in the C-terminal tail (variants analyzed in the current study) were not associated with prostate cancer. This is consistent with the findings that a form of GEN1, lacking the C-terminal, is sufficient for its activity [34, 35]. However, the role of p. Asp514Ilefs*13, p. Lys645Cysfs*29 and p.Lys839Glufs*2 on GEN1 function has not been investigated and needs to be studied.

Testing for germline mutations by next-generation sequencing is important for cancer prevention, surveillance, and the management of cancer patients. Available testing panels comprise genes with strong, limited, or unknown associations with prostate cancer. The current multigene tests include focused, guideline-based, and comprehensive panels. It is challenging to decide which panel to use in a particular clinical situation. In particular, this applies to comprehensive panels containing genes associated with various cancers. *GEN1* is included in several panels (i.e., the Fulgent Full Comprehensive Cancer Panel and the Invitae DNA Damage Repair Panel). In men where a *GEN1* variant is found, the subjects will want to know if they are at increased risk of prostate cancer. Our study suggests that *GEN1* changes are not associated with prostate cancer susceptibility.

## Conclusions

Our findings suggest that *GEN1* is not a prostate cancer susceptibility gene. In this context our study has clinical implications for genetic counseling of men who tested positive for germline variants of *GEN1*.

## Data Availability

No datasets were generated or analysed during the current study.

## Abbreviations

DNA: Deoxyribonucleic acid
HJ: Holliday junction
HR: Hazard ratio
IHCC: International Hereditary Cancer Center
LOH: Loss of heterozygosity
NGS: Next-generation sequencing
OR: Odds ratio
P: Probability value
PC: Prostate cancer
PSA: Prostate-specific antigen
SNP: Single nucleotide polymorphism
UK: United Kingdom
WES: Whole exome sequencing
